# Anteroposterior versus anterolateral pacer pad position in patients with symptomatic bradycardia

**DOI:** 10.1016/j.ijcha.2025.101857

**Published:** 2026-01-06

**Authors:** Andreas Goldschmied, Manuel Sigle, Ioannis Toskas, Mirac Senel, Livia Dingemann, Malte Kranert, Tobias Harm, Meinrad Gawaz, Michal Droppa, Andreas Brendlin, Karin Anne Lydia Mueller

**Affiliations:** a Department of Cardiology and Angiology, University Hospital Tübingen, Eberhard Karls University Tübingen, Tübingen, Germany; b Department of Radiology, University Hospital Tübingen, Eberhard Karls University Tübingen, Tübingen, Germany

**Keywords:** Bradycardia, Transcutaneous pacing, AV-nodal block, Pacemaker device, Clinical study

## Abstract

**Introduction:**

Transcutaneous cardiac pacing (TCP) is an important emergency treatment option in patients with symptomatic bradycardia. With the help of a portable pulse generator an electrical current is delivered through the patient́s thorax in order to induce ventricular contractions. Data on patients in sinus rhythm suggests favorable pacing thresholds when using an anteroposterior (AP) compared to an anterolateral (AL) pacer pad positioning. However, evidence in bradycardic patients is lacking.

**Methods:**

We conducted a prospective crossover clinical study which included 16 patients with symptomatic bradycardia. Patients received consecutive TCP in an AP and AL position under sedoanalgesia. TCP was carried out in an AP and an AL pacer pad position if patients were hemodynamically stable (systolic blood pressure > 90 mmHg). Minimal required current and other variables were noted for both pacer pad positions and Wilcoxon Signed Rank tests were used to compare differences.

**Results:**

We did not overserve a significant difference in minimal required pacing current between the AP and AL pacer pad position (median threshold AP = 125 mA [±48], median threshold AL = 140 mA [±78], p = 0.53). However, a linear mixed-effects model revealed higher pacing thresholds in patients on beta blockers (B = 72.1, p < 0.001, 95 % CI = 36.6–107.7) and with lower myocardial mass (B = -0.41, p < 0.001, 95 % CI = −0.59- −0.23).

**Conclusion:**

We observed no significant difference in pacing thresholds between an AP and AL pacer pad position in patients with symptomatic bradycardia. These results do not align with prior work investigating a monitor with pulsed current delivery.

## 1 Introduction

Transcutaneous cardiac pacing (TCP) is an important emergency treatment option in patients with symptomatic bradycardia. With the help of a portable pulse generator and two electrode pads which are placed on the patient’s skin, an electrical current is delivered through the patient́s thorax in order to depolarize myocardial cells and induce ventricular contractions. In hemodynamically unstable patients, this can be a lifesaving maneuver to bridge until definitive treatment and is recommended in current guidelines [[1], [2], [3], [4], [5], [6]]. In practice, the current between the two pacer pads is increased until myocardial contractions and sufficient blood flow are achieved. This is associated with a similar QRS morphology and hemodynamic response when compared to transvenous pacing [7]. Since the applied current can lead to painful contraction of skeletal muscles and skin burns, sedoanalgesia is required [8]. Since TCP is usually carried out in critically ill and unstable patients, quick ventricular capture and low doses of sedating medication which might lead to further circulatory depression are desired.

Even though guidelines do not provide specific, evidence-based recommendations [9,10], pacer pad position might affect the minimal amount of needed electrical current to achieve ventricular capture. Moayedi et al. demonstrated that an anteroposterior (AP) pacer pad position is associated with lower pacing thresholds when compared to an anterolateral (AL) pacer pad position [11]. They enrolled patients scheduled to undergo elective cardioversion for atrial fibrillation or flutter and conducted TCP after successful cardioversion. However, this patient collective did not suffer from symptomatic bradycardia and would not require TCP in an emergency setting.

We therefore compared an AP to an AL pacer pad position in patients suffering from symptomatic bradycardia. To ensure participant safety, individuals were enrolled after successful permanent pacer implantation and backup pacing frequency was reduced to 35 beats per minute (bpm). Then, TCP was conducted in both positions and minimal required current was noted. Furthermore, other factors that might affect pacing thresholds like laboratory values, echocardiographic or clinical data were analyzed.

## 2 Methods

### 2.1 Study design

**Fig. 1:**
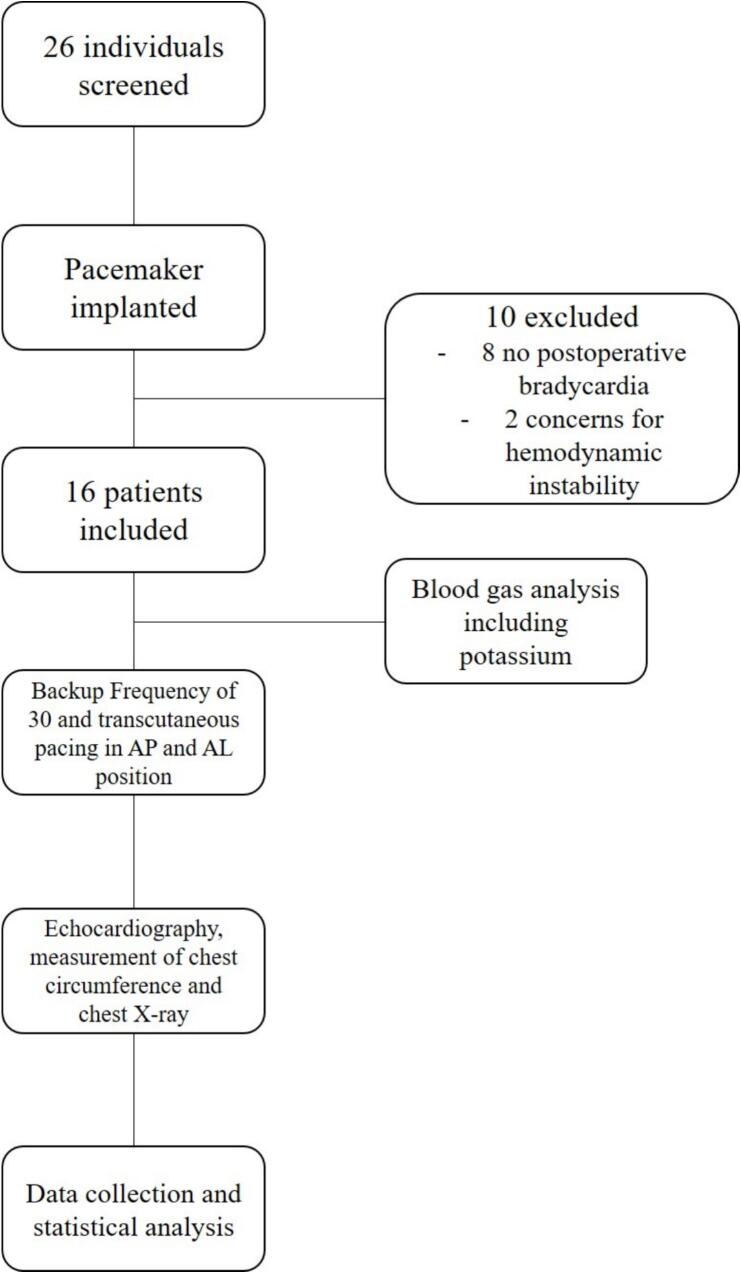
Flowchart of the study design.

This single center prospective crossover clinical study was carried out between August 2023 and February 2025 at the Department of Cardiology and Angiology of the University Clinic Tuebingen, Germany. 16 consecutive patients who presented with symptomatic bradycardia (ischemic chest pain, hypotension, altered mentation, syncope, dizziness) requiring permanent pacemaker implantation were included. Inclusion criteria included age > 18 years, signed written informed consent and bradycardia with a heart rate < 40 bpm after successful permanent pacemaker implantation. Clinical data like age, sex, height, weight, chest circumference as well as data on prior medical history, current medication, laboratory values, echocardiographic and radiological data (chest X-ray) were collected from the digital patient chart. The study was approved by the institutional ethics committee (247/2023BO2) and complies with the declaration of Helsinki and the good clinical practice guidelines [[12], [13], [14]]. A flow chart of the study design is displayed in Fig. 1.

A sample size calculation for analyses of the primary endpoint was carried out based on the results by Moayedi et al. [11]. At an expected relative difference of 26.2 % between the groups, a sample size of 7 patients was estimated to achieve a statistical power of 95 % at a two-sided alpha level of 5 % with the help of the power calculation function of IBM SPSS Statistics Version 28.0 (IBM, Armonk, USA). In order to achieve a sufficient sample size to also investigate the secondary endpoint, we chose to ultimately include a total of 16 patients.

### 2.2 Study protocol

Prior to permanent pacemaker implantation, suitable patients signed written informed consent. Pacer pads (Lifepak Quik-Combo; Physio-Control, Redmond, Washington, USA) were attached in an AP as well as an AL position (after chest hair in the area was removed using a mechanical hair clipper) similar to the study by Moayedi et al. [11]. An arterial line was put into either the right or left radial artery and a pulse oximeter was put on either the left or right index finger. Analgosedation via propofol and piritramide was carried out during permanent pacemaker implantation and TCP. After successful implantation, backup pacing frequency was reduced to 35 bpm. If intrinsic heart rate was lower than 40 bpm and patients were hemodynamically stable (systolic blood pressure > 90 mmHg), study inclusion commenced on the operating table. To avoid any carryover effects, pad placement order was alternated for each included patient (participants with odd participant numbers were initially paced in the AP position whereas individuals with even participant numbers were initially paced in the AL position). The external pacing device (Lifepak 20e; Medtronic, Fridley, Minnesota, USA) was set to synchronous mode and the heart rate to 60 bpm. Starting at a current of 10 mA, the output was steadily increased at a rate of 10 mA per second until the heart rate (measured via the arterial waveform) was 60 bpm for 10 s. The arterial waveform before and after successful electrical capture is depicted in Supplementary Fig. S1. Pulse oximetry was purely used to monitor patients’ oxygen saturation during analgosedation and not to detect electrical capture by TCP. Subsequently, the other set of pads was connected to the external pacing device and the second pacing threshold was determined in the identical way. If capture was not achieved by 200 mA (the pacer’s maximum output), failure to capture was noted. Permanent pacing thresholds were increased according to the treating electrophysiologists clinical judgment and a blood gas analysis was carried out before termination of sedation and removal of the arterial line.

### 2.3 Study objectives

The primary endpoint was the difference in electrical current required for successful TCP between an AP and an AL pacer pad position. Secondary endpoints included analysis of other parameters (laboratory values, clinical, echocardiographic data) on overall pacing thresholds.

### 2.4 Parameter calculation

Body surface area was calculated using the Du Bois formula [15]. Left ventricular (LV) mass was calculated according to current recommendations by the American Society of Echocardiography and the European Association of Cardiovascular Imaging [16]. Chest circumference was measured on mamillar level. Anterior-posterior and lateral diameter of the bony thorax on chest X-ray was measured on sternal level of the 5. intercostal space. In order to estimate soft tissue that might affect electrical current during TCP, thoracic soft tissue distance was calculated by subtracting bony thorax circumference from chest circumference. This value correlated well with patient BMI (*p* = 0.029, CI = 0.053–0.839) (Supplementary Fig. S2). Bony thorax circumference was calculated by applying Ramanujańs formula for estimating circumference of an elliptic body.

### 2.5 Statistical analysis

All statistical analysis were performed using IBM SPSS Statistics Version 28.0 (IBM, Armonk, USA) and R Version 4.3.0. Continuous variables were displayed as medians ± interquartile range and categorial variables were displayed as counts and percentages. A spearman correlation coefficient was calculated to investigate correlation between parameters thoracic soft tissue distance and BMI. Wilcoxon Signed Rank tests were used to compare difference between AP and AL pacing thresholds. If capture could not be achieved in one of the two pacer pad position, a value of 201 mA was assigned to the non-capturing position for analyzes.

For exploring influence on pacing thresholds by other factors then pacer pad position, a linear mixed-effects model with a random intercept for each subject was created. This linear mixed-effects model was fitted using pacing threshold as the dependent variable with pad position, left ventricular mass, beta blocker therapy, thoracic soft tissue distance, serum potassium concentration, and pacing order as fixed effects, and participant identity as a random intercept to account for repeated measures. Full diagnostic assessment of the linear mixed model was performed and is demonstrated in Supplementary Fig. S3. Model assumptions were evaluated by visual inspection of residual diagnostics, including histograms and Q-Q plots to assess normality, and residuals-versus-fitted value plots to assess homoscedasticity. Linearity of continuous variables was examined by plotting residuals against each predictor. Potential outliers were identified using standardized residuals, with all observations retained given the robustness of mixed-effects models to subject-level variability.

A two-sided alpha level of < 0.05 was considered statistically significant for all tests.

### 2.6 Visualization

IBM SPSS Statistics Version 28.0 (IBM, Armonk, USA), Microsoft Power Point (Microsoft Corporation, Redmond, USA) and RStudio (Posit PBC, Boston, USA) package “ggplot2” were used to generate figures.

## 3 Results

### 3.1 Baseline characteristics

Patients in our cohort had a median age of 80 years and about half were male and on beta-blockers. Median BMI was 25.8 kg/m^2^ and median BSA 1.9 m^2^. About one third was anticoagulated with a DOAC. Roughly 40 % of patients were treated with a SGLT2 inhibitor and about half were on ASA. Median left and right ventricular function was normal while median GLS was reduced (− 13.6 %). Median LV mass was 147.5 g and median RV free wall diameter was 5 mm. 6.3 % of patients suffered from COPD and 18.8 % from Asthma while roughly 50 % had CAD. AV nodal block accounted for almost 90 % of bradycardic arrhythmias. Full baseline characteristics are displayed in Table 1.

**Table 1:** Patient characteristics of the study population (n = 16).

Variable	Value
**Clinical data**	
Age (years)	80 (±16)
Gender (male)	9 (56.3)
BMI (kg/m^2^)	25.8 (±4)
BSA (m^2^)	1.9 (±0.4)
Chest circumference (cm)	100 (±10)
Thoracic soft tissue distance (cm)	21 (±17)
**Medication**	
ß-blocker	9 (56.3)
Amiodarone	1 (6.3)
ACE inhibitor	6 (37.5)
ARB	3 (18.8)
ARNI	2 (12.5)
MRA	4 (25)
DOAC	5 (31.3)
SGLT2 inhibitor	7 (43.8)
ASA	9 (56.3)
Platelet aggregation inhibitor	4 (25)
**Echo data**	
Left ventricular EF (%)	60 (±15)
GLS (%)	−13.6 (±9.7)
TAPSE (mm)	21 (±8)
LV Mass (g)	147.5 (±153)
RV free wall diameter (mm)	5 (±2)
**Laboratory values**	
Potassium (mmol/l)	4 (±0.8)
NT-pro BNP (ng/l)	2762 (±3874)
**Electrocardiographic data**	
AV nodal block	14 (87.5)
Bradyarrhythmia	1 (6.3)
No intrinsic rhythm	1 (6.3)
**Patient history**	
Coronary artery disease	9 (56.3)
Arterial hypertension	10 (62.5)
Diabetes	4 (25)
Asthma	3 (18.8)
COPD	1 (6.3)
Chronic kidney disease	2 (12.5)

Continuous variables are displayed as median ± interquartile range and categorial variables are displayed as counts and percentages. With the exception for one missing value for the variable thoracic soft tissue distance, there were no missing data. BMI – body mass index, BSA – body surface area, ACE – angiotensin converting enzyme, ARB – angiotensin receptor blocker, ARNI – angiotensin receptor/neprilysin inhibitor, MRA – mineralocorticoid receptor antagonist, DOAC – direct oral anticoagulant, SGLT2 – sodium/glucose cotransporter 2, ASA – acetylsalicylic acid, EF – ejection fraction, GLS – global longitudinal strain, TAPSE – tricuspid annular plane systolic excursion, RV – right ventricle, NT-pro BNP − N-terminal prohormone of brain natriuretic peptide, AV – atrioventricular, COPD – chronic obstructive pulmonary disease.

### 3.2 Required current was slightly lower in an AP position in patients with symptomatic bradycardia

**Fig. 2:**
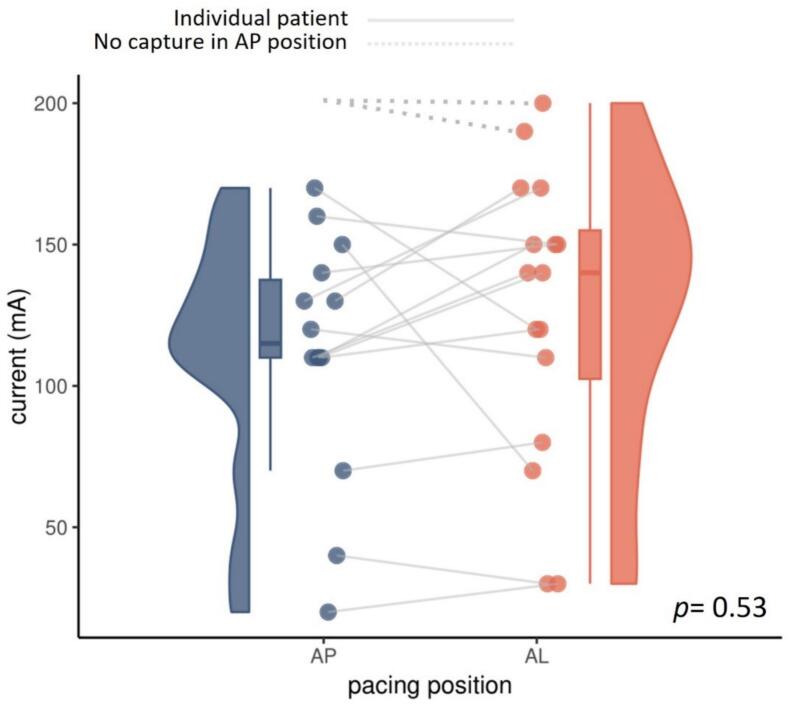
**Paired boxplots demonstrating pacing thresholds in the AP and AL position.** Pacer pad position is displayed on the x-axis while minimal required current is displayed on the y-axis. Lines connect individual patients while doted lines are used in individuals where no pacing could be achieved in one pacer pad position and a value of 201 mA had to be assigned.

As mentioned in the methods section, we conducted paired analyzes to explore difference in pacing threshold between the AP and AL position. Although median minimal required current is slightly lower in the AP position, there was no significant difference in our statistical analyses (median threshold AP = 125 mA [±48], median threshold AL = 140 mA [±78], p = 0.53). Paired boxplots demonstrating pacing threshold in both positions are demonstrated in Fig. 2. Possible ordering effects are displayed in Supplementary Table S1.

### 3.3 Beta blockers and LV mass influence pacing threshold

**Fig. 3:**
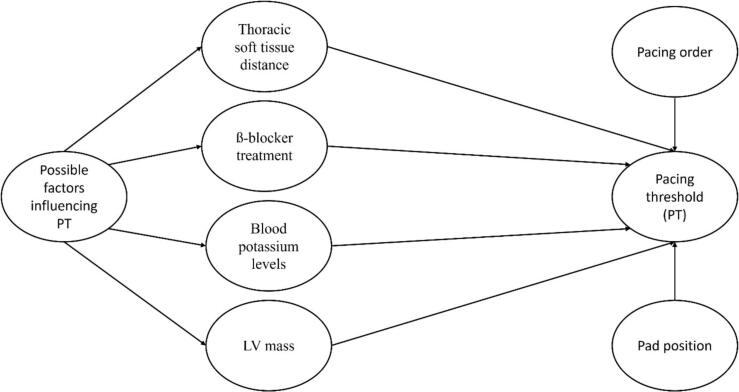
Directed acyclic graph demonstrating the linear mixed-effects model.

In order to identify further influences on pacing threshold (n = 16) we conducted a linear mixed-effects model. A directed acyclic graph showing model development is presented in Fig. 3. To investigate a broad spectrum of possible interactions, a laboratory parameter (blood potassium), an echocardiographic parameter (LV mass), a radiographic parameter (thoracic soft tissue distance) and a clinical parameter (beta blocker treatment) were selected. From the four investigated variables, absence of therapy with beta blockers (B = 72.1, p < 0.001, 95 % CI = 36.6–107.7) and increase in LV mass (B = -0.41, p < 0.001, 95 % CI = -0.59- −0.23) were associated with lower pacing thresholds. These associations occurred independent of pacer pad position and pacing order (AP then AL versus AL then AP) (Table 2).

**Table 2:** Linear mixed-effects model to predict pacing threshold.

Significant variables	B	SE	*p*-value	95 % CI	
				LB	UB
Beta blocker	72.1	18.14	**<0.001**	36.56	107.69
LV mass	−0.41	−0.09	**<0.001**	−0.59	−0.23
Potassium	−11.48	30.64	0.708	−71.53	48.56
Tissue distance	−0.67	1.10	0.539	−2.82	1.47
Pad position	−0.53	8.37	0.949	−16.94	15.88
Pacing order	3.89	23.76	0.870	−42.69	50.46

B – regression coefficient, SE – standard error, CI – confidence interval, LB – lower bound, UB – upper bound.

**Fig. 4:**
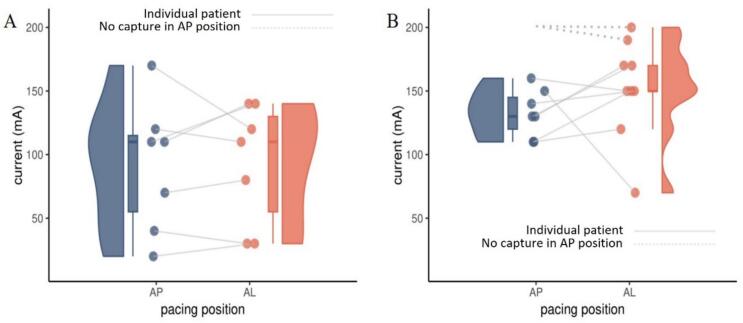
**Difference in pacing threshold depending on pacer pad position stratified to beta blocker therapy.** Pacing position is shown on the x-axis while pacing current is demonstrated on the y-axis in patients without (**A**) and with (**B**) beta blocker therapy.

Pacing thresholds in the AP and AL position stratified to the strongest predictor of pacing threshold (beta blocker treatment) are demonstrated in Fig. 4. As mentioned above, our linear mixed-effects model demonstrated higher pacing thresholds in the beta blocker group independent of pacer pad position (B = 72.1, p < 0.001, 95 % CI = 36.6–107.7). In 32 measurements, capture could not be achieved twice. Both instances occurred in the AP position in patients with beta blocker treatment. As stated in the methods section, 201 mA was assigned to these non-capturing positions for statistical analyzes.

## 4 Discussion

In our prospective crossover clinical study, minimal current needed to achieve myocardial contractions was slightly lower when using an AP position but the difference was little and not statistically significant. However, our linear mixed-effects model demonstrated that a lack of beta blocker treatment and increase in myocardial mass is associated with lower pacing thresholds.

There could be several reasons why we were not able to reproduce the results by Moayedi et al. despite using a similar study design. Most importantly, the patient collective differed considerably. While they included patients after cardioversion into a sinus rhythm, we specifically investigated a cohort of individuals with symptomatic bradycardia requiring implantation of a permanent pacemaker.

Despite our best efforts, our study population did differ from individuals requiring emergency TCP in clinical practice. Even though bradycardic, the investigated population was hemodynamically stable while TCP was performed. At the moment, we cannot state whether or not our findings are translatable to patients requiring emergency TCP. Such a setting is difficult to replicate in a study and would likely require a multicenter approach and possible inclusion of EMS. Furthermore, carrying out a study under highly critical circumstances would raise considerable ethical questions regarding patient safety.

Besides the difference in cardiac rhythm, the two study cohorts differed in several other aspects: our cohort was older, more likely to suffer from CAD, had a higher BMI but was less likely to have a past medical history of asthma and COPD. Since laboratory values, echocardiographic data and information on medication is not provided for the patients investigated by Moayedi et al., we cannot make a statement regarding those variables.

Compared to Moayedi et al., pacing threshold was higher in our collective (93 mA vs. 125 mA) for AP and (126 mA vs. 140 mA) for the AL position. This results in a higher median pacing threshold in our collective which might in part be explained by an increase in fibrotic tissue and impairment in electrical conduction in older individuals, especially in the presence of bradycardic conduction disturbances [17].

Considering the increase in pacing threshold was more pronounced in the AP position, this explanation alone seems to be insufficient. AV nodal block, which accounted for almost 90 % of conduction abnormalities in our collective, forces subsidiary pacemaker cells to generate escape rhythms and maintain cardiac function [18]. This changes myocardial electrophysiologic axis and could limit the beneficial effects of the AP position observed by Moayedi et al.

Another possible factor, is the presence of a permanent pacemaker in our collective. In order to provide patient safety, individuals were included after a permanent pacemaker system was in place to ensure a safe backup frequency. In theory, systems are shielded against external electrical and magnetic fields [19,20] but we cannot fully exclude bias by the permanent pacemaker system.

When planning the present study, we did a sample size calculations based on the available data by Moayedi et al. [11]. Since we did observe a small benefit in the AP group, our collective might just be underpowered to demonstrate a significant effect in patients with symptomatic bradycardia. A post-hoc analyses of the achieved power was carried out and demonstrated a very limited power for the small observed difference in pacing thresholds (0.064) However, if present, it appears that this small effect is negligible in clinical practice.

In our collective, we observed low rates of failure to pace (2 out of 32 measurements). This might be explained by using up to 200 mA compared to a maximum of 140 mA by Moayedi et al. Since both events occurred in the AP position, we cannot confirm former reports of lower failure to capture rates in this subgroup [21].

Furthermore, the device used by Moayedi et al. provided by Zoll medical (Chelmsford, Massachusetts, USA) uses a 40-millisecond constant current pulse which is unique compared to other companies. This is supposed to increase capture rates and lower pacing thresholds [22]. As mentioned before, average reported pacing currents were indeed lower but we cannot confirm higher rates of capture using this technique. It is important to note, that due to these differences in used device, our findings might not be translatable to defibrillators using a 40-millisecond constant current pulse.

Another important difference is the methodology regarding confirmation of electrical capture. While Moayedi et al. used pulse oximetry, we relied on direct confirmation with an invasively measured arterial waveform. Since there is data suggesting “false electrical capture” is common in clinical practice [23], we feel like this is the best method to reliably confirm successful TCP. Even though unlikely, muscle contractions could generate pulsatile venous flow, potentially mimicking successful capture on pulse oximetry. Of note, confirmation of electrical capture can also be achieved with the help of point of care echocardiography in clinical practice [24,25].

As a secondary endpoint, influence of clinical factors, laboratory values, echocardiographic data and patient medication on pacing thresholds were examined. Medical therapy with beta blockers proved to be a strong predictor of high pacing thresholds in TCP. This can be explained by the druǵs negative dromotropic and chronotropic properties and is an entirely novel finding since there has been no evidence on an influence of beta blockers on permanent pacemaker thresholds [26,27].

Surprisingly, thoracic soft tissue distance did not affect pacing thresholds in our linear regression model. Under the premise that more soft tissue between pacer pads and the heart could increase the required current, we used radiologic and clinical data in order to specifically quantify this variable (see methods sections).

To our knowledge, this is the first study linking an increase in myocardial mass to lower pacing thresholds. More available myocardium could facilitate myocardial depolarization if the current is applied transcutaneous and not directly to the heart via pacemaker leads.

We cannot exclude other factors influencing transcutaneous pacing thresholds. Especially blood potassium levels have been associated with multiple effects on myocardial conduction but did not affect pacing thresholds in our analysis [[28], [29], [30]]. However, our population did not include individuals with abnormal levels of blood potassium so we cannot make assumptions regarding this patient population.

## 5 Limitations

There are several limitations in our study. Our sample size might have been too small in order to detect significant benefits of an AP pacer pad position. Furthermore, pacing thresholds were observed by increasing pacing currents at 10 mA increments. Although reflecting clinical practice, this results in our outcome not being a true continuous variable. Furthermore, despite our best efforts, our study collective differed from patients in need of emergency TCP in that they were not truly hemodynamical unstable and pacemaker leads had been implanted. Also, data on prior transvenous pacing or application of chronotropic agents was not available. This makes translatability to clinical practice uncertain.

## 6 Conclusion

We did not observe a significant difference in pacing threshold between an AP and AL pacer pad position (median threshold AP = 125 mA [±48], median threshold AL = 140 mA [±78], p = 0.53) but treatment with beta blockers (B = 72.1, p < 0.001, 95 % CI = 36.6–107.7) and low myocardial mass (B = -0.41, p < 0.001, 95 % CI = -0.59- −0.23) required higher electrical currents to provide TCP in bradycardic patients.

Based on this data, we cannot recommend a specific pacer pad position in TCP but higher currents should be expected in the presence of beta blocker treatment or low myocardial mass.

## 7 Author contribution statement

A.G., and K.M. designed and planned the study. A.G., I.T., M.S., L.B., T.H. and M.K. collected the data. A.B. collected and analyzed X-Ray data, A.G. analyzed the data including statistical analysis. AG. and M.S. designed the figures. A.G. and K.M. wrote and edited the manuscript. M.G. and M.D. revised the manuscript. All authors participated in the interpretation and discussion of the results.

## 8 Data availability

Study data is available from the corresponding author upon reasonable request.

## CRediT authorship contribution statement

**Andreas Goldschmied:** Writing – review & editing, Writing – original draft, Visualization, Validation, Methodology, Investigation, Formal analysis, Data curation. **Manuel Sigle:** Writing – review & editing, Writing – original draft, Visualization, Validation, Methodology, Investigation, Formal analysis. **Ioannis Toskas:** Writing – review & editing, Visualization, Investigation. **Mirac Senel:** Writing – review & editing, Validation, Investigation. **Livia Dingemann:** Writing – review & editing, Validation, Investigation. **Malte Kranert:** Writing – review & editing, Validation, Investigation. **Tobias Harm:** Writing – review & editing, Visualization, Validation. **Meinrad Gawaz:** Writing – review & editing, Supervision, Resources, Project administration, Methodology. **Michal Droppa:** Writing – review & editing, Validation, Supervision. **Andreas Brendlin:** Writing – review & editing, Validation, Investigation. **Karin Anne Lydia Mueller:** Writing – original draft, Visualization, Validation, Supervision, Project administration, Methodology, Formal analysis, Data curation, Conceptualization.

## Informed consent

Written informed consent was obtained in all patients participating in the study.

## Ethics approval

The study complies with the declaration of Helsinki and good clinical practice guidelines and was approved by the responsible local ethics committee (project number 247/2023B02).

## Funding

None.

## Declaration of competing interest

The authors declare that they have no known competing financial interests or personal relationships that could have appeared to influence the work reported in this paper.
