# Supplementary material for: Anteroposterior versus anterolateral pacer pad position in patients with symptomatic bradycardia
Source: Int J Cardiol Heart Vasc. 2026 Jan 6;62:101857. doi: 10.1016/j.ijcha.2025.101857 (PMC13153136; doi:10.1016/j.ijcha.2025.101857)
Supplement: Supplementary Data 1 [file mmc1.docx]

**Anteroposterior versus anterolateral pacer pad position in patients with symptomatic bradycardia**

**Short title:** Pad position of external pacer matters?

Andreas Goldschmied^1^, MD, Manuel Sigle^1^, MD, Ioannis Toskas^1^, MD, Mirac Senel^1^, MD, Livia Dingemann^1^, MD, Malte Kranert^1^, MD, Tobias Harm^1^, MD, Meinrad Gawaz^1^, MD, Michal Droppa^1^, MD, Andreas Brendlin^2^, MD, Karin Anne Lydia Mueller^1#^; MD

^1^Department of Cardiology and Angiology, University Hospital Tübingen, Eberhard Karls University Tübingen, Tübingen, Germany

^2^Department of Radiology, University Hospital Tübingen, Eberhard Karls University Tübingen, Tübingen, Germany

# corresponding author:

Karin Anne Lydia Mueller

Department of Cardiology and Angiology, University Hospital Tübingen, Eberhard Karls University Tübingen

Otfried‐Müller-Str. 10, 72076 Tübingen, Germany

E-Mail: [K.Mueller@med.uni-tuebingen.de](mailto:K.Mueller@med.uni-tuebingen.de)

**Supplementary material**

Supplementary Figure S1

Supplementary Figure S2

Supplementary Figure S3

Supplementary Table S1


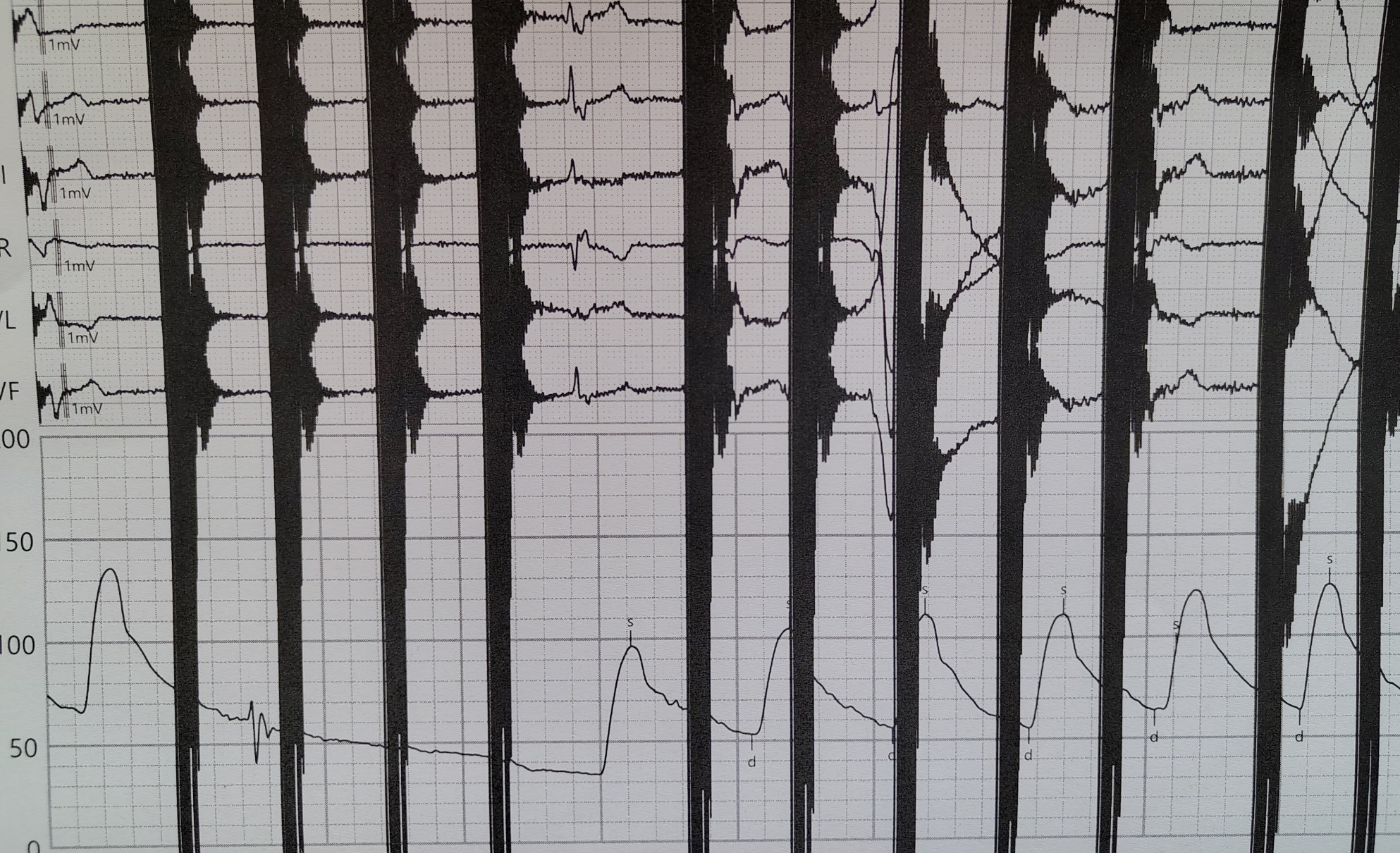


**Supplementary figure S1:** Arterial waveform before and after successful capture using TCP.


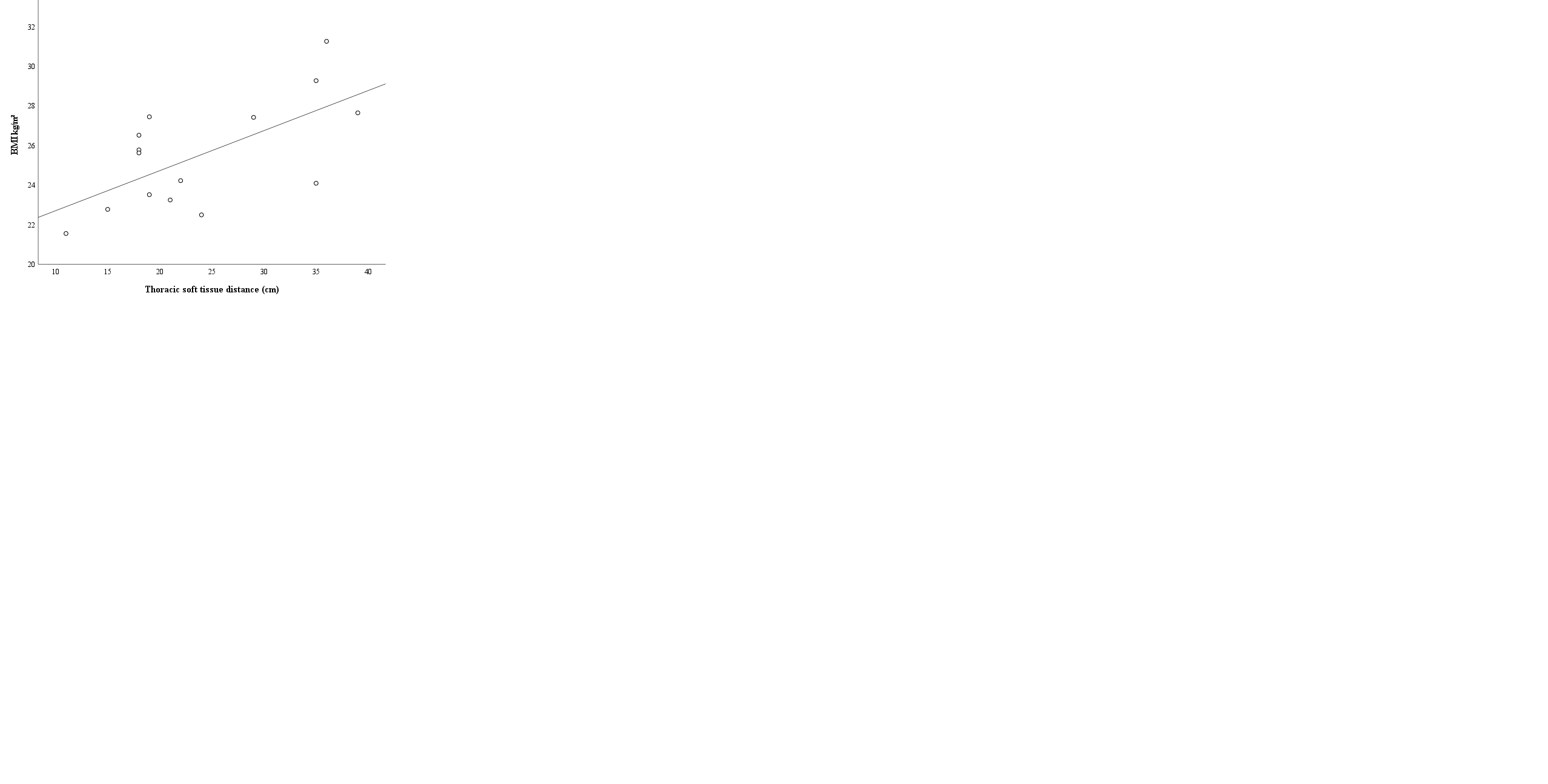


*p= 0.029*

**Supplementary figure S2:** Correlation between parameters thoracic soft tissue distance and BMI (BMI – body mass index).


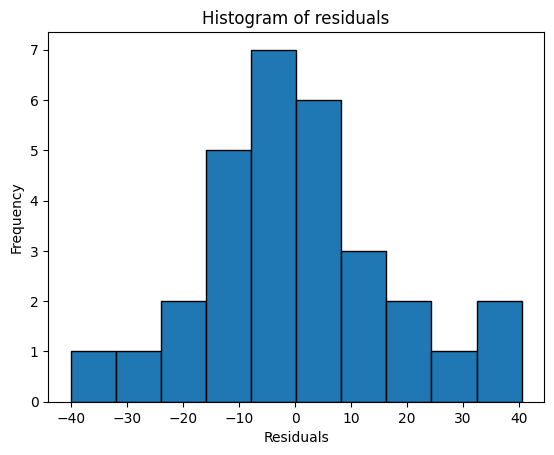


**A**


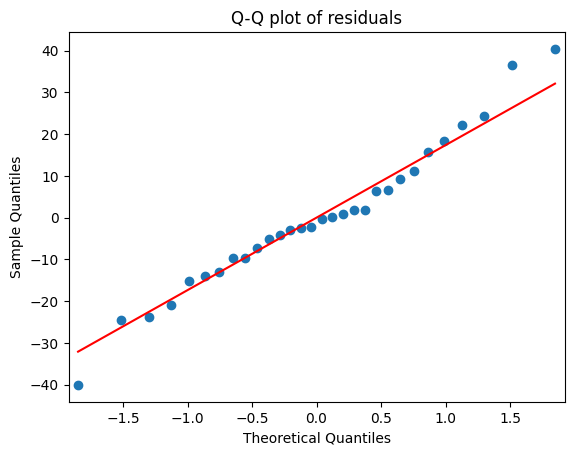


**B**


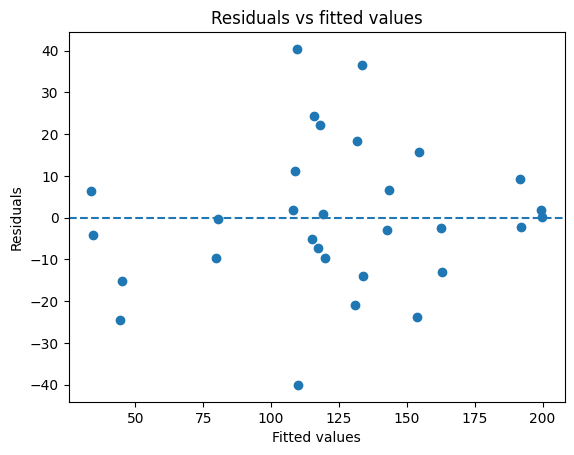


**C**


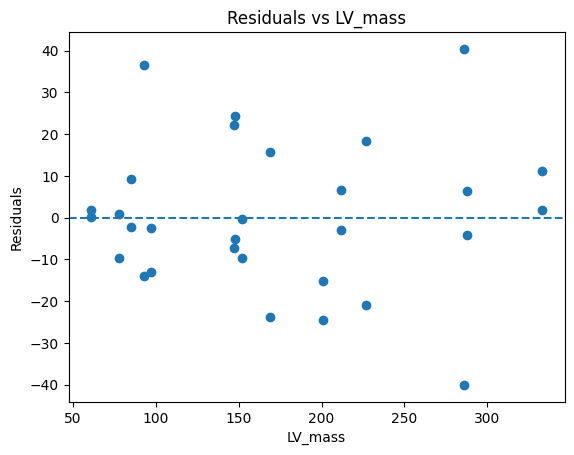


**D**


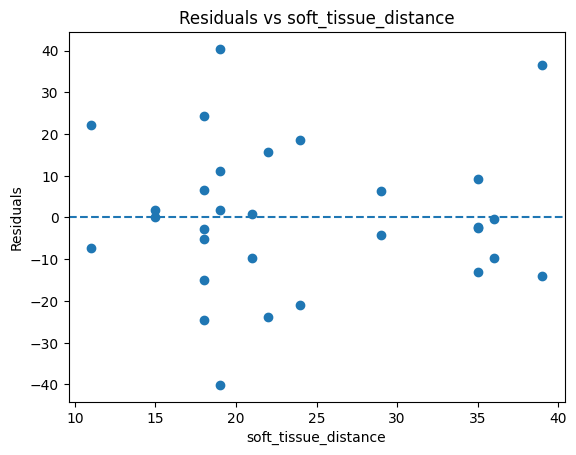


**E**


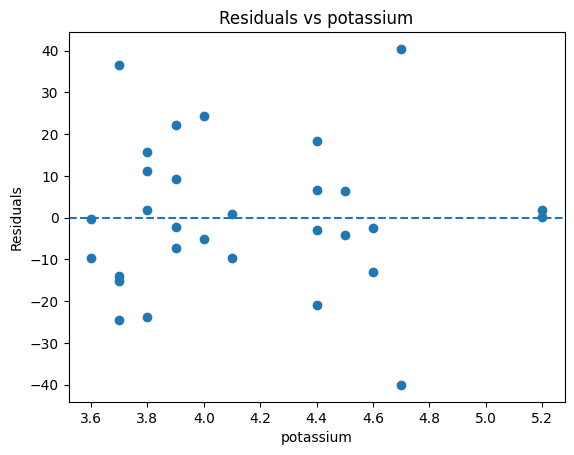


**F**

**Supplementary figure S3:** Full diagnostic assessment of the linear mixed model. The residuals were approximately normally distributed (**A**) with acceptable deviation at extremes based on the histogram and q-q plot (**B**). Scatterplots (**C-F**) of residuals versus fitted values and predictor variables did not demonstrate patterns suggestive of heteroscedasticity or non-linearity.

**Supplementary table S1: Ordering effects of the primary endpoint**

|  | **Pacing pad position** | |  |
| --- | --- | --- | --- |
| **Pacing sequence** | **1** | **2** | **Within individual difference: AL-AP** |
| **AL then AP** |  |  |  |
| Median (IQR) | 135 (85) | 140 (55) | -10 (70) |
| Sample size | 8 | 8 | 8 |
| **AP then AL** |  |  |  |
| Median (IQR) | 110 (58) | 140 (75) | 10 (20) |
| Sample size | 8 | 8 | 8 |
| **Effect** |  |  |  |
| Median (IQR) and CI | - | - | 10 (40); CI: -10.5-20 |
| Sample size | - | - | 16 |
| Wilcoxon Signed Rank tests | - | - | 0.53 |

IGR – Interquartile range, CI - Confidence interval
